# 4391 Big data analysis of adolescent obesity, pregnancy and kidney function

**DOI:** 10.1017/cts.2020.163

**Published:** 2020-07-29

**Authors:** Dana Bielopolski, Neha Singh, Caroline Jiang, Robert Bruce MacArthur, Kimberly Vasquez, Dena Moftah, Rhonda G Kost, Jonathan N. Tobin

**Affiliations:** 1 Rockefeller University; 2 Clinical Directors Network, Inc. (CDN)

## Abstract

OBJECTIVES/GOALS:

1. Examine the associations among BMI and markers of cardiometabolic risk, including blood pressure, lipids and blood glucose.
2. Assess prevalence of kidney function deterioration, identified as hyperfiltration and moderately increased albuminuria (MIA), in obese compared to normal weight adolescents.

METHODS/STUDY POPULATION: De-identified electronic health records (EHR) data were extracted for female adolescents, aged 12-21 years, and their offspring through 24 months, who received health care services (Jan 2012 to Dec 2016) in NYC from 12 academic health centers and community health centers that are part of PCORnet NYC Clinical Data Research Network (NYC-CDRN). Data were analyzed using SAS (version 3.2.5). Patient characteristics overall and for study subgroups were examined using standard summary statistics. Trends in cardio-renal variables were examined by BMI groups coded according to NHANES as underweight, normal weight, overweight or obese. Multiple linear regression analyses will control for covariates. RESULTS/ANTICIPATED RESULTS: Data from 651,066 adolescent females ages 12-21 were retrieved. Analysis was performed on a subset of 202,214 unique patients (26% white, 15% black, 12.9% Latina) for whom there was complete data for BMI and blood pressure. Distribution of BMI was 6% underweight, 59% normal weight, 19% overweight, and 17% obese. There were significant differences in mean systolic (SBP, mean±SD mmHg: 102±12, 108±11, 112±12, 116±12) and diastolic blood pressure (DBP, mean±SD mmHg: 62±10, 66±8, 68±8.9, 70±9) across the four BMI groups with an increasing trend (p-values<0.0001). We will examine renal function trends, and whether these cardio-renal differences persist when controlling for age, race and ethnicity. DISCUSSION/SIGNIFICANCE OF IMPACT: Although SBP/DBP means were within normal limits across BMI groups, significant increasing trends suggest that women in higher BMI groups may be at increased risk for hypertension and potentially for renal dysfunction. We will examine contributions of race/ ethnicity and age to these associations.

